# Draft Genome Sequence for *Ralstonia* sp. Strain OR214, a Bacterium with Potential for Bioremediation

**DOI:** 10.1128/genomeA.00321-13

**Published:** 2013-06-06

**Authors:** Sagar M. Utturkar, Annette Bollmann, Ryann M. Brzoska, Dawn M. Klingeman, Slava E. Epstein, Anthony V. Palumbo, Steven D. Brown

**Affiliations:** Graduate School of Genome Science and Technology, University of Tennessee, Knoxville, Tennessee, USAa; Department of Biology, Northeastern University, Boston, Massachusetts, USAb; Department of Microbiology, Miami University, Oxford, Ohio, USAc; Biosciences Division, Oak Ridge National Laboratory, Oak Ridge, Tennessee, USAd

## Abstract

*Ralstonia* sp. strain OR214 belongs to the class *Betaproteobacteria* and was isolated from subsurface sediments in Oak Ridge, TN. A member of this genus has been described as a potential bioremediation agent. Strain OR214 is tolerant to various heavy metals, such as uranium, nickel, cobalt, and cadmium. We present its draft genome sequence here.

## GENOME ANNOUNCEMENT

*Ralstonia* spp. are Gram-negative, nonfermentative, rod-shaped bacteria that are ubiquitous in water and soil (1). These bacteria are able to live in oligotrophic environments and thrive under harsh environmental conditions (2). *Ralstonia* spp. have been shown to have the capability to biodegrade multiple organic compounds, including toxic aromatic hydrocarbons (3, 4), carcinogenic groundwater pollutants, chlorophenol compounds from pesticides, nitroaromatics, and quinoline compounds from manufacturing industries (5). *Ralstonia* spp. are candidates for bioremediation, as they offer several advantages, including the ability to break down a variety of organic compounds, and they may have potential for widespread environmental use due to their oligotrophic nature. *Ralstonia* spp. have been used for the remediation of toluene from groundwater in South Carolina (6).

*Ralstonia* sp. strain OR214 was isolated from subsurface sediments from the Field Research Center (FRC) in Oak Ridge, TN, which were acidic and contaminated with heavy metals and organic pollutants (7). *Microbacterium laevaniformans* strain OR221, another bacterium tolerant to metals, nitrate, and low pH conditions, also isolated from the FRC site, recently had its genome sequence determined (8). Strain OR214 is tolerant to high concentrations of heavy metals, such as up to 500 mM of nitrate, 200 µM of uranium, 500 µM of nickel, 50 µM of cobalt, and 50 µM of cadmium, and to a lowest pH of 3.5 (7). We generated the draft genome sequence of strain OR214 to gain insights into its physiology.

Draft genome sequence data for strain OR214 were generated using a combination of 454 FLX (9) and Illumina MiSeq (10) technologies with single-end and 500-bp paired-end libraries, respectively. The 454 data consisted of 595,223 reads and generated 194,042,698 bp. After quality trimming of Illumina data (CLC Genomics Workbench, version 5.5.1), there were 571,527,000 bp of sequence data, with an average read length of 132 bp. Trimmed Illumina reads were assembled with CLC Genomics Workbench, and consensus sequences were fragmented into 1.5-kbp overlapping fake reads using the fb_dice.pl script from the FragBlast module (http://www.clarkfrancis.com/codes/fb_dice.pl). The Newbler application (version 2.6, 454; Life Sciences) was used to assemble the fragmented Illumina consensus and 454 reads into 46 large (≥500 bp) contigs, with a total genome size of 5.4 Mb. The N_50_ contig size is 321,662 bp, with the largest contig being 632,308 bp, and the genome has an overall estimated G+C content of 63.4%.

The draft genome was annotated at the Joint Genome Institute (JGI) Integrated Microbial Genomes (IMG) database and comparative analysis system (11), which predicted 5,305 candidate protein-encoding gene models for *Ralstonia* sp. OR214. A number of predicted heavy metal-sensing proteins, heavy metal-translocating efflux pumps, and monooxygenase genes potentially involved in the breakdown of aromatic compounds were identified in the genome, which may facilitate its resistance in harsh environments. Genome sequences for *Ralstonia* sp. 12D and 12J, which were resistant to high concentrations of heavy metals, are also available through IMG. The strain OR214 draft genome sequence will allow for the comparison of biodegradation genes across different strains and contribute toward bioremediation research.

### Nucleotide sequence accession numbers.

This Whole-Genome Shotgun project has been deposited at DDBJ/EMBL/GenBank under the accession no. APMQ00000000. The version described in this paper is the first version, accession no. APMQ00000000 .

## References

[1] Gilligan PH Lum G Vandamme P Whittier S 2003 *Burkholderia*, *Stenotrophomonas*, *Ralstonia*, *Brevundimonas*, *Comamonas*, *Delftia*, *Pandoraea*, and *Acidovorax*, p 729–748 *In* Murray PR Baron EJ Jorgensen JH Pfaller MA Yolken RH (ed), Manual of clinical microbiology, 8th ed American Society for Microbiology, Washington, DC

[2] McAlister MB Kulakov LA O’Hanlon JF Larkin MJ Ogden KL 2002 Survival and nutritional requirements of three bacteria isolated from ultrapure water. J. Ind. Microbiol. Biotechnol. 29:75–82 12161774 10.1038/sj.jim.7000273

[3] Kukor JJ Olsen RH 1990 Molecular cloning, characterization, and regulation of a *Pseudomonas pickettii* PKO1 gene encoding phenol hydroxylase and expression of the gene in *Pseudomonas aeruginosa* PAO1c. J. Bacteriol. 172:4624–4630 2115872 10.1128/jb.172.8.4624-4630.1990 PMC213297

[4] Kukor JJ Olsen RH 1992 Complete nucleotide sequence of *tbuD*, the gene encoding phenol/cresol hydroxylase from *Pseudomonas pickettii* PKO1, and functional analysis of the encoded enzyme. J. Bacteriol. 174:6518–6526 1400204 10.1128/jb.174.20.6518-6526.1992 PMC207615

[5] Ryan MP Pembroke JT Adley CC 2007 Ralstonia pickettii in environmental biotechnology: potential and applications. J. Appl. Microbiol. 103:754–764 17897177 10.1111/j.1365-2672.2007.03361.x

[6] Vroblesky DAR JF Petkewich MD Chapelle FH Bradley PM Landmeyer JE 1997 Remediation of petroleum hydrocarbon-contaminated ground water in the vicinity of a jet-fuel tank farm, Hanahan, South Carolina. U.S. Geolog. Survey Water Res. Investig. Rep. 1997 61:96–4155

[7] Bollmann A Palumbo AV Lewis K Epstein SS 2010 Isolation and physiology of bacteria from contaminated subsurface sediments. Appl. Environ. Microbiol. 76:7413–7419 20870785 10.1128/AEM.00376-10 PMC2976195

[8] Brown SD Palumbo AV Panikov N Ariyawansa T Klingeman DM Johnson CM Land ML Utturkar SM Epstein SS 2012 Draft genome sequence for *Microbacterium laevaniformans* strain OR221, a bacterium tolerant to metals, nitrate, and low pH. J. Bacteriol. 194:3279–3280 22628508 10.1128/JB.00474-12 PMC3370866

[9] Margulies M Egholm M Altman WE Attiya S Bader JS Bemben LA Berka J Braverman MS Chen YJ Chen Z Dewell SB Du L Fierro JM Gomes XV Godwin BC He W Helgesen S Ho CH Ho CH Irzyk GP Jando SC Alenquer ML Jarvie TP Jirage KB Kim JB Knight JR Lanza JR Leamon JH Lefkowitz SM Lei M Li J Lohman KL Lu H Makhijani VB McDade KE McKenna MP Myers EW Nickerson E Nobile JR Plant R Puc BP Ronan MT Roth GT Sarkis GJ Simons JF Simpson JW Srinivasan M Tartaro KR Tomasz A Vogt KA Volkmer GA 2005 Genome sequencing in microfabricated high-density picolitre reactors. Nature 437:376–380 16056220 10.1038/nature03959 PMC1464427

[10] Loman NJ Misra RV Dallman TJ Constantinidou C Gharbia SE Wain J Pallen MJ 2012 Performance comparison of benchtop high-throughput sequencing platforms. Nat. Biotechnol. 30:434–439 22522955 10.1038/nbt.2198

[11] Markowitz VM Chen IM Palaniappan K Chu K Szeto E Grechkin Y Ratner A Jacob B Huang J Williams P Huntemann M Anderson I Mavromatis K Ivanova NN Kyrpides NC 2012 IMG: the integrated microbial genomes database and comparative analysis system. Nucleic Acids Res. 40:D115–D122 22194640 10.1093/nar/gkr1044 PMC3245086

